# A case report of an accidental iatrogenic dexmedetomidine overdose in an adult

**DOI:** 10.1186/s12245-024-00613-5

**Published:** 2024-02-27

**Authors:** Suvi-Maria Tiainen, Jonni Unga, Panu Uusalo

**Affiliations:** 1 Department of Anaesthesiology and Intensive Care, University of Turku, Kiinamyllynkatu 4-8, P.O. Box 51, Turku, FI-20521 Finland; 2 Division of Perioperative Services, Intensive Care and Pain Medicine, Turku University Hospital, Kiinamyllynkatu 4-8, P.O. Box 51, Turku, FI-20521 Finland; 3 Department of Anaesthesiology and Intensive Care, Satasairaala Central Hospital, Satakunta Hospital District, Pori, Finland; 4 Department of Emergency Medicine, University of Turku, Kiinamyllynkatu 4-8, P.O. Box 51, Turku, FI- 20521 Finland

**Keywords:** Alpha2-agonists, Medication error, Adverse effect, Oversedation, Case report

## Abstract

**Background:**

Dexmedetomidine is a sedative drug with a wide safety margin.

**Case presentation:**

We present a case of accidental iatrogenic dexmedetomidine overdose in an adult patient during high-intensity focused ultrasound (HIFU) treatment. This is the first case report of an adult patient receiving an intravenous push of dexmedetomidine. Overdose resulted in severe oversedation, but symptoms receded spontaneously over time.

**Conclusions:**

Dexmedetomidine overdoses are infrequent, and they are usually the result of an administration error.

## Introduction

The alpha-2 adrenoceptor agonist dexmedetomidine is commonly used for sedation in intensive care units and operating rooms. Dexmedetomidine has a large margin of safety, and overdoses typically occur due to a dosing error.

There are a few earlier case reports discussing dexmedetomidine overdose: a small case series on accidental perioperative overdose in three adult patients [1] and three case reports about overdose in pediatric patients [2–4].

We report an accidental overdose of dexmedetomidine in an adult patient. Our case is the first to describe an overdose in an adult patient, where undiluted dexmedetomidine was administered as a rapid intravenous push. As in most of these previously reported cases, the reported overdose did not cause significant life-threatening problems, and symptoms resolved spontaneously during surveillance. We also provide a literature review and a summary of previously published reports on dexmedetomidine overdose. This case report follows the CARE (CAse REport) guidelines [5].

## Case presentation

A 68-year-old 95 kg (209 pounds) and 184 cm (6 feet) male with a body mass index (BMI) of 28.1 kg/m^2^ was scheduled for high-intensity focused ultrasound (HIFU) treatment to treat essential tremor (a nervous system condition that causes involuntary and rhythmic shaking).

The patient had been fasting before the procedure. Before the HIFU treatment commenced, the patient received oral acetaminophen, oral pantoprazole, oral etoricoxib, intravenous betamethasone, and intravenous ondansetron as premedication. The procedure is performed under magnetic resonance imaging and occurs in the radiology department. It is done in cooperation with neurosurgeons, radiologists, neurologists, and HIFU nurses. The patient received standard monitoring, including pulse oximetry and telemetry. Local infiltration anesthesia with ropivacaine 5 mg/ml 10 ml and lidocaine cum adrenaline 10 mg/ml 10 ml was administered to spots on the scalp, where a custom frame was fitted in order to keep the patient’s head still during the procedure. Additional local infiltration of 15 ml lidocaine 10 mg/ml was added later. No sedation is given during the procedure as the neurologist examines the patient and the treatment response.

The HIFU treatment was given as planned. After the treatment, the subject was supposed to be given 10 mg of intravenous dexamethasone (5 mg/ml, 2 ml vial). However, the wrong vial was chosen, and the subject received one vial (2 ml) of intravenous dexmedetomidine (100 mcg/ml) instead. Thus, the subject received a 200 mcg bolus of undiluted intravenous dexmedetomidine. The error was unrecognized at this point.

A few minutes after the end of treatment, the subject suddenly fell asleep. He was drowsy but could be easily woken up. Shortly, his consciousness began to decrease further. The subject had difficulties keeping his eyes open, and his speech began to slur. The neurologist and neurosurgeon attended to examine the patient. The patient’s vital functions remained steady (RR 127/72 mmHg, pulse rate 59/min, SpO2 97%), but his consciousness decreased further. After a short while, the subject did not respond to any stimuli. His pupils were symmetrical and constricted. He was breathing spontaneously, but apnea periods lasting 10–20 s were noticed.

The subject was transferred to the emergency department for further examination. Upon arrival in the emergency department, blood glucose was 10.7 mmol/l (192.6 mg/dL), the temperature was 35.3 °C (95.54 F), and there was a sinus rhythm in the ECG. Because medication error was still not noticed at this point, the possibility of cerebral hemorrhage was suspected, and the subject received a full stroke protocol, including head computed tomography (CT), which was revealed to be normal. Approximately 60 min after dexmedetomidine administration, it was discovered that the patient had accidentally received dexmedetomidine instead of dexamethasone. Since the patient was hemodynamically stable, his respiratory parameters, including arterial blood gas analysis, were normal, and his GCS began to increase slowly; a “watchful waiting” approach was chosen based on a short (2.0-2.5 h) half-life of dexmedetomidine [6]. The situation was resolved by itself during the observation. After five hours, the patient had regained full consciousness. He had an uneventful recovery and was discharged the next day. The vital parameters of the patient are presented in Table 1.

**Table 1:** Vital parameters at different time points after dexmedetomidine administration

Timepoint	BP (mmHg)	HR (1/min)	RR (1/min)	SpO2 (%)	GCS
Baseline	142/84	82	-	-	15
10 min	127/72	59	13	97	10
1 h	129/80	56	19	94	3
2 h	112/71	56	18	95	14
3 h	102/70	51	16	97	15
6 h	101/60	59	16	95	15

BP = blood pressure; HR = heart rate; RR = respiratory rate; SpO2 = peripheral oxygen saturation; GCS = Glasgow Coma Scale

## Discussion and conclusions

This is the first case report describing dexmedetomidine overdose in an adult patient, where dexmedetomidine was administered as a rapid intravenous push. Table 2 presents the characteristics of the previous case reports on dexmedetomidine overdose.

**Table 2:** Previous case reports on dexmedetomidine overdose

Author	Publication	Year	Study type	Patient(s)	Environment	Dexmedetomidine dosage	Overdose symptoms	Treatment
Jorden et al. [1]	Annals Pharmacotherapy	2004	Case-series	1. 74-year-old male, 68 kg 2. 51-year-old male, 95 kg 3. 29-year-old male, 214 kg	1. adjunct to general anesthesia 2. ICU sedation 3. ICU, weaning from respirator	1. intravenous infusion (total 2,8 mcg/kg) 2. intravenous infusion 2–4 mcg/kg/h (total 19 mcg/kg) 3. intravenous infusion 0.5 mcg/kg/min (total 10 mcg/kg)	1. mild hypotension and bradycardia 2. deep sedation 3. deep sedation	1. observation 2. observation 3. observation
Bernard et al. [4]	J Clin Anesthesia	2009	Case-report	20-month-old female, 10.7 kg	cardiac catheterization to close a patent ductus arteriosus	intravenous infusion 1 mcg/kg/min (total 36 mcg/kg)	hypoglycemia, hypertension, deep sedation	glucose correction, observation
Nath et al. [3]	Indian J Anesthesia	2013	Case-report	3-year-old male, 11 kg	pyogenic meningitis	100mcg as an intravenous bolus (total 9mcg/kg)	hypotension, miosis, profound sedation, bradypnea	oxygen supplementation by Venturi mask, saline boluses, adrenaline infusion
Li et al. [2]	Pediatric Anesthesia	2019	Case-report	23-month-old male, 12 kg	magnetic resonance imaging and neurosurgery	intravenous infusion 0.1–0.2 mcg/kg/min (total 22mcg/kg)	initial hypertension, bradycardia, hypotension	noradrenaline infusion

The recommended dosing for dexmedetomidine is an intravenous maintenance infusion of 0.2–0.7 mcg/kg/h. Additionally, a loading dose of 1 mcg/kg over 10 min may be given to shorten the onset of action. Doses in the previous case reports on dexmedetomidine overdose exceeded the recommended infusion rates by 10 to 60-fold. In all earlier reported cases except one by Nath et al., dexmedetomidine administration occurred as a slow infusion. Nath et al. hypothesized that the administration rate is the most significant factor determining the degree of overdose symptoms. When dexmedetomidine is administered as a bolus or as an intravenous push, symptoms seem more pronounced than after slow infusion, although the actual dose per kilogram may be significantly higher in infusion. Our findings support this assumption because the patient’s symptoms were considerable even though the dose per kilogram (2.1 mcg/kg) was relatively small compared with other reports.

Dexmedetomidine is usually considered a mild sedative, and it is known to produce a state of cooperative sedation at average infusion rates. However, the sedative effect of dexmedetomidine is dose-dependent, and plasma levels above 1.9 ng/mL are thought to cause significant sedation [7]. Our patient was deeply sedated, similar to patients in most of the previously described cases.

The most frequent side effects of dexmedetomidine (even with recommended dosages) are hypotension and bradycardia. Usually, reductions in heart rate are mild. Dexmedetomidine has a biphasic hemodynamic effect: lower plasma concentrations induce sympatholysis-mediated hypotension, whereas higher plasma concentrations induce vasoconstriction and hypertension. Both hypotension and hypertension are prevalent symptoms in earlier presented overdose cases, but our patient remained surprisingly stable, although the peak plasma concentrations of dexmedetomidine must have reached quite high levels. Compared to most other sedative agents, dexmedetomidine has minimal effects on respiration [8]. However, at high doses, bradypnea is possible.

In one of the previous case reports, hypoglycemia was reported after dexmedetomidine overdose [4]. On the contrary, our patient had high blood glucose (considering he had been fasting). Dexmedetomidine’s effect on blood glucose is complex. On the other hand, pancreatic beta-cells are regulated by alpha2-adrenoreceptors, and alpha2-agonists such as dexmedetomidine directly suppress insulin secretion. However, dexmedetomidine also lowers cortisol levels, which may result in lower blood glucose levels.

The primary treatment for dexmedetomidine overdose is supportive care. Dexmedetomidine has a short elimination half-life, approximately two hours. In all of the presented cases, the overdose symptoms resolved within a few hours. Although dexmedetomidine overdosing situations are relatively uncommon and usually manageable with supportive care, an antidote would enhance safety. A specific antidote for dexmedetomidine exists already. However, it is not approved for use in human patients. Atipamezole is a synthetic α2 adrenergic receptor antagonist that is used to reverse the sedative effect of alpha-agonists in animals. It is routinely used to reverse sedation and hasten recovery rapidly after veterinary procedures. Atipamezole has been studied in humans, and it has proven to be effective [9, 10]. However, since atipamezole is not approved for humans, it is not readily available in emergency departments.

Most previously documented cases of dexmedetomidine overdose resulted from infusion pump programming errors. In this case, however, an error was a consequence of look-alike and sound-alike (LASA) drugs, dexamethasone and dexmedetomidine. According to estimates, up to 25% of pharmaceutical errors are caused by LASA [11]. One possible solution to prevent this kind of error is using tall man lettering (TML), which is a method for distinguishing medicine names that look alike by using capital letters (dexAMETHasone and dexmedeTOMIDine).

Dexmedetomidine overdoses usually present with deep sedation and hemodynamic disturbances, but other symptoms, such as hypoglycemia, are also possible. The situation is typically self-resolving, and treatment of dexmedetomidine overdose consists mainly of supportive care.

## Data Availability

No datasets were generated or analysed during the current study.
